# Relationships Between Sleep Problems and Stress Coping Strategies Adopted by Nurses Including Socio-Occupational Factors

**DOI:** 10.3389/fpsyt.2021.660776

**Published:** 2021-06-10

**Authors:** Krystyna Kowalczuk, Elżbieta Krajewska-Kułak, Marek Sobolewski

**Affiliations:** ^1^Department of Integrated Medical Care, Medical University of Bialystok, Bialystok, Poland; ^2^Faculty of Management, Rzeszow University of Technology, Rzeszow, Poland

**Keywords:** nurse, stress, sleep, insomnia, excessive sleepiness, coping strategies

## Abstract

**Introduction:** The health of nurses has a direct impact on the quality of care and health outcomes for patients. The length and quality of sleep as well as the intensity of perceived stress have an impact on the health of nurses. An appropriate stress coping strategy can reduce the impact of stress and mitigate its negative consequences.

**Aim:** The purpose of this study was to investigate relationships between excessive sleepiness and insomnia in interaction with selected socio-occupational factors and stress coping strategies among nurses.

**Material and Method:** The cross-sectional study was conducted among 448 nurses working in hospitals in Podlasie Province in Poland. Mini-Cope inventory - the polish adaptation of Carver's BriefCope was used for measuring coping with stress. Sleep problems were assessed using the Athens Insomnia Scale and the Epworth Sleepiness Scale.

**Results:** The most frequent used coping strategies were active strategies (*active coping, planning*). The least-used were avoidant strategies (*behavioral disengagement, substance use*). Excessive sleepiness affected 38% of surveyed, while insomnia 33%. Excessive sleepiness was most strongly related with *behavioural disengagement* (*R*^2^ = 18.6%), *substance use* (*R*^2^ = 17.5%), *humour* (*R*^2^ = 13.8%) and *denial* (*R*^2^ = 12.0%) while insomnia with *substance use* (*R*^2^ = 17.5%) and *self-blame* (*R*^2^ = 15.9%). Nurses with tertiary education experiencing sleep problems less frequently used the strategy of *humour, behavioural disengagement, substance use* and *religion* than with lower education. Nurses working in interventional wards experiencing excessive sleepiness used the strategy of *humour, religion* and *positive reframing* less often than those working in other wards while those suffering from insomnia used the strategy of *humour* more often than those working in other wards.

**Conclusions:** The implementation of avoidant and support-seeking and emotion-oriented stress coping strategies by nurses were associated with the sleep problems. Tertiary education discourages nurses with sleep problems from using avoidant coping strategies and devoting themselves to religion. Education and improvement of the quality of sleep may prevent nurses from resorting to stress coping strategies that have a detrimental effect on their health and well-being.

## Introduction

Nursing is one of the most stressful jobs in the world. Research carried out in Poland by an employers' organization places nursing in the second group of the most stressful occupations (1), whereas in the USA, nursing is in the top five most stressful occupations according to the scale developed by the Occupational Information Network (2). In studies conducted among Polish nurses, from 73.6 to 95.6% of respondents stated that the profession of a nurse is stressful (3, 4). In addition, even 56.1% of respondents claimed that they are exposed to work stress every day (5).

In the occupational setting, the exposure of nurses to stress and the constant need to express empathy while providing care to patients are inherent elements of daily work (6, 7). Factors influencing the perception of occupational stress by nurses include shift work, interpersonal conflicts with other staff members, role conflict, lack of support from doctors, low levels of autonomy as well as low control over their job, rotation between hospital units, and aggressive behaviour of co-workers and patients (8). In addition, stress is intensified because of daily contacts with seriously ill patients, and dealing with death and dying (9, 10). The association between stress and negative health outcomes is well-documented. Stress can alter human homeostasis and physiological or hormonal balance that are highly associated with health problems. Long-term exposure of nurses to stress has a negative effect on mental health, and also leads to behavioural problems (11, 12). Furthermore, longitudinal research among nurses has documented a robust association between stress and chronic diseases (13, 14). Adverse health behaviours resulting from occupational stress include sleep disorders, smoking, alcohol and drug abuse, and also absenteeism from work (15). Prolonged exposure to stressors can also lead to fully symptomatic burnout syndrome and depression (16). The effectiveness of nurses' coping techniques affects their health and well-being. Nurses experiencing high levels of stress and, at the same time, not actively coping with stress had the worst health outcomes and the most risky behaviors to health compared to other nurses (17, 18).

Each response to stress is a subjective appraisal and depends on the psychophysiological status of a person, the nature of the stressor, the type of environment where exposure to stress takes place, age, level of education, and knowledge of coping strategies (19). Importantly, personal perception of stress changes with age, workload, acquired occupational experience, and other coexisting problems, for instance at home that modify the person's view of the existing or resolved problems.

Coping with stress is considered the main element of the general stress process. Studies have revealed that an adequate way of coping can reduce the impact of stress and mitigate its negative consequences. Many theories on coping strategies have been coined over the years (20). Lazarus and Folkman defined coping as *a phenomenon that involves both cognitive and behavioural responses that individuals use in an attempt to manage internal and/or external stressors perceived to exceed their personal resources* (21). In this approach, coping is a targeted effort made by an individual if he or she identifies the situation to be stressful. Lazarus defined two functions of coping with stress: instrumental, and based on self-adjustment. The purpose of instrumental or task-oriented coping is to solve the problem and improve the situation by modifying personal destructive behaviour, or by changing the environment that poses a threat. The second function, self-adjustment, involves controlling negative emotions, or stimulation which mobilizes for action (22).

Endler and Parker identified three strategies for coping with stress: task-oriented, emotion-oriented and avoidant (23, 24). Task-oriented coping is used by people with high emotional intelligence and high social competencies. It is chosen by people who have the resources to make efforts to solve problems and change the existing situation. Emotion-oriented coping is characterized by focusing on the self and personal negative emotions in a stressful situation. Avoidant coping is characteristic of people who tend to avoid thinking about or experiencing stressful situations. There are two subtypes of avoidant coping: avoidant-distracted and avoidant-social. The first one means people have recourse to distracting behaviours (e.g., binge eating, drowsiness, thinking about pleasant things, reading books or watching TV more often than usual), and the second strategy means seeking contact with other people (25).

The vast majority of Polish nurses working in hospitals work in a 12-h shift system (8). There are many studies documenting the relationship between shift work and sleep disorders (26, 27). Shift work is in conflict with the human biological clock. A conventional daily schedule matching the biological clock ensures that people have good performance and conserve energy necessary for physiological processes. Shift work, including night work, can cause a permanent dysregulation of the sleep-wake cycle. Some nurses working shifts cannot adapt to the inversed rhythm of sleep and wakefulness (28). After night shifts, sleeping during daytime is difficult and does not provide optimal rest. Nurses often prefer to sleep off night shifts at night during non-working days rather than sleep during the day after night shifts (29). Stress-induced changes in sleep-architecture may contribute to cognitive dysfunction and psychiatric disorders (30).

There are a number of studies analysing the relationship between stress and sleep problems. The relationship between the poor quality of sleep and stress is bidirectional (17) but most of the available studies have focused on investigating the effect of stress on sleep quality. Most of them report a negative effect of stress on the quality and quantity of sleep, and also excessive sleepiness (31, 32). The inverse relationship between stress and sleep problems is much less studied and is generally limited to the impact of insomnia on occupational stress levels. Strategies for coping with stress appear in such studies as moderating factors (33, 34).

The individual's coping style has been rarely addressed in sleep and stress studies. The first study that has explored this issue was a retrospective study of sleep and stress coping style conducted with college students (35). Findings of this study indicated that short sleepers used more emotion oriented strategies than long sleepers. Morin (18) reported that, in comparison with controls, insomnia patients relied more on emotion oriented coping strategies and perceived their lives as more stressful. Sadeh and Gruber (36) suggest that avoidant coping styles would be more likely to lead to excessive sleepiness (escape to sleep). Increased sleep is likely to reduce exposure to stressors and the associated adverse feelings and cognitions. Therefore, emotion oriented coping is more likely to produce physiological arousal and disrupt or reduce sleep (37).

There is very little research focusing on the direct relationship of sleep problems and adopted stress coping strategies. In particular, there is a lack of such studies carried out among nurses—the professional group described as one of the most stressed and mostly doing shift work.

The mental health of nurses has a direct impact on the quality of care and health outcomes for patients (38, 39). The length and quality of sleep as well as the intensity of perceived stress have an impact on the health of nurses (40–42). An appropriate stress coping strategy can reduce the impact of stress and mitigate its negative consequences. Therefore, the aim of the study was to investigate the relationships between insomnia and excessive sleepiness independently and in interaction with socio-occupational factors such as age, type of hospital ward, education and frequency of sick leaves, and the strategies of coping with stress adopted by nurses. The vast majority of Polish hospital nurses are women mostly working in a 12-h shift system, therefore the interaction with gender and the work system was not considered in our study.

## Materials and Methods

The cross-sectional study was conducted from September to December 2019, in Białystok, Poland. It involved registered nurses working in hospitals in Podlasie Province. Participation in the study was voluntary, and all procedures were approved by the Bioethical Commission of the Medical University of Bialystok, ref. no R-I-002/332/2019.

### Study Group Selection

The selection of respondents for the study group was based on the registry of nurses associated in the District Chamber of Nurses and Midwives in Białystok. The total number of registered nurses was 6,085 (5,990 women and 95 men). The selection criterion was work based on employment contract in a hospital. Nurses working part-time and on other than employment contract were excluded.

### Study Protocol

The study used was conducted using paper-based questionnaires. The questionnaires were distributed by researchers to the nurses during courses organized by the District Chamber of Nurses and Midwives in Białystok. Participation was voluntary. Before the study, each nurse was informed about the anonymity of the conducted research, and about the possibility of withdrawing from the study without stating a reason. They were asked to complete the surveys in their free time within 2 weeks and send the completed questionnaires in a sealed envelope to the investigators' address. Six hundred questionnaire surveys were distributed, out of which 448 correctly completed questionnaires were returned. The response rate was 75%. There are no known reasons why 152 respondents did not participate in the study. All the demographic and occupational data were obtained from surveys in the form of respondents' self-reports. No incentives were used to encourage participation in the study.

### Description of the Questionnaire and the Applied Measures

The research instrument for measuring coping with stress was the Mini-COPE multidimensional inventory (43), a Polish version of BriefCOPE—abbreviated version of Carver's COPE inventory (44). Carver designed her multidimensional COPE inventory based on the Lazarus model of stress (21) and the model of behavioural self-regulation proposed by Scheier and Carver (45). The Mini-COPE inventory measures 14 identified coping responses. They could be grouped into three key stress management strategies: active coping, avoidant coping, and support-seeking/emotion-oriented coping. Active strategy includes following responses: *active coping, planning and positive reframing*. Avoidant strategy includes: *acceptance, humour, self-distraction, denial, substance use and behavioural disengagement* and Support-seeking and emotion-oriented strategy includes: *religion, use of emotional support, use of instrumental support, venting and self-blame*. Each response can be scored from 0 (a response is not used at all) to 3 (a response often used). These measures were standardized by the authors of the questionnaire. The original BriefCOPE inventory and its Polish version of Mini-COPE were extensively validated, and both have clear scoring guidelines. Cronbach's alpha coefficients for individual scales range from 0.62 (for *Venting* strategy) to 0.89 (for *Religion* strategy).

Insomnia was assessed using the Athens Insomnia Scale (AIS). This is a self*-*report questionnaire consisting of eight statements regarding various insomnia symptoms and uses diagnostic criteria from the International Classification of Diseases (ICD-10). These symptoms are rated by the respondent on a 0–3 scale, where 0 means no problem and 3 means a very serious problem. The total score on the AIS is from 0 to 24. Scores not higher than 5 indicate no problems with sleep, scores from 6 to 10 indicate the risk of insomnia, and scores higher than 11 indicate existing insomnia. The questionnaire was validated by the authors of the Polish version (46). The internal consistency measured using Cronbach's alpha coefficient was 0.91—higher than reported by authors of Polish version.

The Epworth Sleepiness Scale (ESS) was used to determine the occurrence of excessive sleepiness. The ESS developed by Johns is a self-report instrument to assess respondent's likelihood of falling asleep in 8 hypothetical situations in daily life. Scores lower than 10 indicate no propensity to sleepiness, and scores higher than 10 indicate excessive sleepiness that should result in a consultation with a doctor. The ESS questionnaire has been validated many times in different countries. The internal consistency measured using Cronbach's alpha coefficient was 0.85—one of the highest value in comparison to other studies (47).

### Analysis

In the descriptive part we presented characteristics of the analysed sample in tables containing the percentage distribution of selected variables or the values of selected descriptive statistics for numerical variables (mean with 95% confidence interval, median, standard deviation).

The relationship between measures of stress coping strategies (Mini-COPE) and measures of sleep problems (AIS, ESS) we analysed by calculating the Spearman's rank correlation coefficient (*r*_S_) and testing the statistical significance of the relationship.

To determine the relationship between the behaviour of nurses in stressful situations determined by the MiniCOPE measures and the occurrence of sleep problems and socio-occupational factors, 14 regression models were constructed. In these models, the dependent variables were subsequent measures in the Mini-COPE and the set of independent variables consisted of sleep problems measures (AIS, ESS) and selected socio-occupational factors: level of education, age, type of hospital ward and the use of sick leave. We used stepwise regression to select the optimal models in which we included only statistically significant variables. These models are presented and interpreted further in the paper.

## Results

### Study Group

The research group consisted of 448 nurses. The vast majority of the respondents were women (85.7%). The mean age in the studied group was 35.8 years, with a slightly lower median of 32 years. The youngest nurse was 20 years old and the oldest was 61. Every fourth person surveyed was not older than 26 years, and every fourth person was not <47 years old. Over 69.5% of the respondents had completed tertiary nursing education, 13.6% had secondary education with a specialization, and 8.9% had secondary education. The majority of the respondents (57.6%) were employed on interventional wards (surgical, intensive care).

### Coping With Stressful Situations and Intensity of Sleep Problems Among Nurses

Descriptive statistics (mean with 95% confidence interval, median, standard deviations) for answers to the Mini-COPE questionnaire and sleep problems questionnaires are presented in Table 1. Excessive sleepiness troubled 37.5% of surveyed nurses. 32.8% of the surveyed nurses complained about insomnia, and 37.1% were at risk of insomnia. The classifications of the intensity of sleep problems were made in accordance with the guidelines for individual questionnaires. It should be emphasized, however, that all further analyses were performed for the numerical measures AIS, ESS and Mini-COPE.

**Table 1:** Descriptive statistics for measures of coping strategies and sleep problems among nurses (*N* = 448).

	**Psychometric measures**	**Mean (95% c.i.)**	**Median**	**Standard deviation**
**Stress coping strategies (MiniCOPE responses)**				
1	Active coping	1.88 (1.81–1.95)	2.0	0.77
2	Planning	1.85 (1.79–1.92)	2.0	0.71
3	Use of emotional support	1.79 (1.72–1.87)	2.0	0.78
4	Use of instrumental support	1.79 (1.72–1.86)	2.0	0.74
5	Acceptance	1.74 (1.68–1.80)	2.0	0.67
6	Positive reframing	1.68 (1.61–1.74)	1.5	0.67
7	Self-distraction	1.59 (1.53–1.66)	1.5	0.70
8	Venting	1.41 (1.35–1.48)	1.5	0.72
9	Religion	1.34 (1.25–1.42)	1.5	0.89
10	Self-blame	1.28 (1.21–1.36)	1.0	0.80
11	Humour	1.12 (1.05–1.20)	1.0	0.77
12	Denial	0.98 (0.91–1.06)	1.0	0.81
13	Behavioural disengagement	0.98 (0.91–1.05)	1.0	0.74
14	Substance use	0.74 (0.66–0.82)	0.5	0.86
**Sleep problems measures**				
	AIS	8.7 (8.2–9.2)	8.0	5.3
	ESS	9.3 (8.8–9.8)	9.0	5.3

### Relation Between Sleep Problems and Coping Strategies

We assessed the correlation between the intensity of sleep problems among nurses and strategies of coping with stress by determining Spearman's rank correlation coefficients for the AIS, ESS scores and the Mini-COPE responses. Results are presented in Table 2.

**Table 2:** Correlations between coping responses and measures of sleep problems (*N* = 448).

**Mini-COPE**	**AIS (score)**	**ESS (score)**
**Active strategies**		
Active coping	−0.06 (0.2163)	0.07 (0.1470)
Planning	−0.03 (0.5127)	0.13 (0.0058^**^)
Positive reframing	−0.02 (0.7384)	0.14 (0.0038^**^)
**Support-seeking and emotion-oriented strategies**		
Religion	0.09 (0.0520)	0.21 (0.0000^***^)
Use of emotional support	−0.08 (0.1064)	0.17 (0.0004^***^)
Use of instrumental support	−0.05 (0.2769)	0.17 (0.0005^***^)
Venting	0.18 (0.0001^***^)	0.34 (0.0000^***^)
Self-blame	0.27 (0.0000^***^)	0.35 (0.0000^***^)
**Avoidant strategies**		
Acceptance	0.03 (0.5352)	0.15 (0.0018^**^)
Humour	0.14 (0.0031^**^)	0.23 (0.0000^***^)
Self-distraction	0.14 (0.0037^**^)	0.25 (0.0000^***^)
Denial	0.22 (0.0000^***^)	0.32 (0.0000^***^)
Substance use	0.25 (0.0000^***^)	0.32 (0.0000^***^)
Behavioural disengagement	0.29 (0.0000^***^)	0.34 (0.0000^***^)

*Statistically significant correlations was highlighted (*

** *p < 0.01*,

*** *p < 0.001)*.

### Sleep Problems and Coping Strategies in Interaction With Socio-Professional Factors

We analysed the relationships between sleep problems, socio-professional factors and the behaviour of nurses in stressful situations. For this purpose we constructed 14 regression models, in which dependent variables were subsequent items of the Mini-COPE inventory divided into three groups of strategies (active, support-seeking and emotion-oriented, avoidant), and the set of independent variables consisted of measures of sleep problems (AIS, ESS), age, level of education, hospital ward and sick leaves. Interactions effects between all pairs of independent variables were included in analysis. We used a stepwise regression to select the optimal models in which we included only statistically significant variables.

#### Sleep Problems and the Use of Active Strategies in Interaction With Socio-Professional Factors

The use of active strategies is more frequent with increasing excessive sleepiness (ESS) and less frequent with increasing insomnia (AIS), except for *planning*. Moreover, it is worth noting that having tertiary education influences more frequent *planning*, and in the case of a *positive reframing*, the impact of ESS on this measure is weaker among people with tertiary education. Moreover, the influence of ESS on the use of *active coping* is more pronounced in the case of work in the interventional wards, and in the case of *positive reframing*, the opposite situation occurs. The models presented in the Table 3 explain from 2.1 to 5.1% of the variability in the frequency of using active strategies in the studied group of nurses.

**Table 3:** Regression models for active strategies (*N* = 448).

**Active strategies**	**Independent variables**	***R*^**2**^**	***B* (95% c.i.)**	**β**	***P***
Active coping	AIS (score)		−0.022 (−0.039; −0.006)	−0.15	0.0073^**^
	ESS (score)	4.6%	0.020 (0.004; 0.037)	0.14	0.0137^*^
	Use of sick leave		0.160 (0.013; 0.307)	0.11	0.0332^*^
	Interventional unit × ESS (score)		0.035 (0.006; 0.064)	0.12	0.0173^*^
Planning	ESS (score)	2.1%	0.018 (0.004; 0.032)	0.13	0.0104^*^
Positive reframing	AIS (score)	5.1%	−0.019 (−0.034; −0.004)	−0.14	0.0145^*^
	ESS (score)		0.032 (0.017; 0.048)	0.24	0.0001^***^
	Tertiary education × ESS (score)		−0.029 (−0.056; −0.001)	−0.11	0.0436^*^
	Interventional unit × ESS (score)		−0.026 (−0.052; 0.000)	−0.10	0.0474^*^

*R2, coefficient of determination; B, regression coefficient with 95% c.i., p-value for significance of each regression coefficient; β, standardize regression coefficient*.

* *p < 0.05*,

** *p < 0.01*,

*** *p < 0.001*.

#### Sleep Problems and the Use of Support-Seeking and Emotion-Oriented Strategies in Interaction With Socio-Professional Factors

More frequent use of support-seeking and emotion-oriented strategies is associated with higher measure of excessive sleepiness (ESS), and is less common among people with higher insomnia measure (AIS) with the exception of *self-blame*, which increases with the AIS measure. Having tertiary education influences the more frequent *use of instrumental support*. The interaction of education with AIS consists in the fact that in nurses with tertiary education, the *religion* strategy occurs less frequently with the increase of the AIS measure. Less frequent use of *self-blame* strategy is influenced by working in the interventional wards and taking a sick leave, in addition, working in the interventional wards reduces the impact of ESS on the frequency of using the *religion* strategy. The models presented in the Table 4 explain from 4.5 to 15.9% of the variability in the frequency of using the searching and emotional strategies in the studied group of nurses.

**Table 4:** Regression models for support-seeking and emotion-oriented strategies (*N* = 448).

**Support-seeking and emotion-oriented strategies**	**Independent variables**	***R*^**2**^**	***B* (95% c.i.)**	**β**	***P***
Religion	ESS (score)		0.031 (0.014; 0.048)	0.18	0.0003^***^
	Tertiary education × AIS (score)	6.9%	−0.044 (−0.079; −0.009)	−0.12	0.0134^*^
	Interventional unit × ESS (score)		−0.041 (−0.075; −0.006)	−0.11	0.0199^*^
Use of emotional support	AIS (score)	4.5%	−0.027 (−0.043; −0.010)	−0.18	0.0015^**^
	ESS (score)		0.035 (0.019; 0.052)	0.23	0.0000^***^
Use of instrumental support	Tertiary education	6.4%	0.236 (0.065; 0.408)	0.14	0.0070^**^
	AIS (score)		−0.016 (−0.033; 0.000)	−0.11	0.0470^*^
	ESS (score)		0.036 (0.020; 0.052)	0.25	0.0000^***^
Venting	ESS (score)	10.6%	0.046 (0.033; 0.058)	0.33	0.0000^***^
Self-blame	AIS (score)	15.9%	0.021 (0.004; 0.037)	0.13	0.0124^*^
	ESS (score)		0.047 (0.031; 0.063)	0.30	0.0000^***^

*R2, coefficient of determination; B, regression coefficient with 95% c.i., p-value for significance of each regression coefficient; β, standardize regression coefficient*.

* *p < 0.05*,

** *p < 0.01*,

*** *p < 0.001*.

#### Sleep Problems and the Use of Avoidant Strategies in Interaction With Socio-Professional Factors

All avoidant strategies are used more frequently as the measure of excessive sleepiness increases. Insomnia affects the more frequent *substance use* and the adoption of *behavioural disengagement* strategy. Having tertiary education reduces the frequency of using the strategy of *denial* and *behavioural disengagement*. Moreover, in the case of using the *humour* strategy, there are interactions of AIS and ESS measures with the education and type of ward. In people with tertiary education, the influence of ESS on the frequency of using the *humour* strategy is weaker than in other people, similarly in the case of the AIS measure. On the other hand, those working in the interventional wards use the *humour* strategy more often with the increase of AIS, and less often with the increase of ESS. The models presented in the Table 5 explain from 2.1 to 18.6% of the variability in the frequency of using the searching and emotional strategies in the studied group of nurses.

**Table 5:** Regression models for avoidant strategies (*N* = 448).

**Avoidant strategies**	**Independent variables**	***R*^**2**^**	***B* (95% c.i.)**	**β**	***P***
Acceptance	ESS (score)	2.1%	0.019 (0.006; 0.032)	0.14	0.0038^**^
Humour	ESS (score)		0.042 (0.028; 0.056)	0.28	0.0000^***^
	Tertiary education × AIS (score)		−0.045 (−0.078; −0.012)	−0.15	0.0075^**^
	Tertiary education × ESS (score)	13.8%	−0.051 (−0.084; −0.018)	−0.17	0.0025^**^
	Interventional unit × AIS (score)		0.044 (0.013; 0.076)	0.15	0.0059^**^
	Interventional unit × ESS (score)		−0.032 (−0.063; 0.000)	−0.11	0.0469^*^
Self-distraction	ESS (score)	6.6%	0.036 (0.023; 0.049)	0.26	0.0000^***^
Denial	Tertiary education	12.0%	−0.178 (−0.352; −0.004)	−0.10	0.0446^*^
	ESS (score)		0.051 (0.036; 0.065)	0.32	0.0000^***^
Substance use	AIS (score)	17.5%	0.032 (0.015; 0.048)	0.19	0.0002^***^
	ESS (score)		0.041 (0.024; 0.058)	0.25	0.0000^***^
	Tertiary education × AIS (score)		−0.058 (−0.089; −0.028)	−0.17	0.0002^***^
Behavioural disengagement	AIS (score)		0.019 (0.004; 0.033)	0.13	0.0141^*^
	ESS (score)	18.6%	0.044 (0.029; 0.060)	0.32	0.0000^***^
	Tertiary education		−0.162 (−0.314; −0.009)	−0.10	0.0377^*^
	Tertiary education × ESS (score)		−0.035 (−0.062; −0.008)	−0.12	0.0103^*^

*R2, coefficient of determination; B, regression coefficient with 95% c.i., p-value for significance of each regression coefficient; β, standardize regression coefficient*.

* *p < 0.05*,

** *p < 0.01*,

*** *p < 0.001*.

## Discussion

Our study revealed that nurses most often used active coping strategies (first, second and sixth place in the ranking), and least often resorted to avoidant strategies (the last four places in the ranking).

Studies conducted among nurses in different countries have provided a spectrum of findings. Similarly to our research, active coping strategies were reported most frequently in studies conducted in Ethiopia (48), Poland (49), Australia and New Zealand (50). Avoidant strategies were least common in Malaysia (51), Ethiopia (48), Poland (49) and Norway (52), what is also consistent with our findings. Different results were reported from study conducted among nurses in India (53) and other study in Malaysia (54), where support-seeking and emotion-oriented strategies were most common, and from studies in Australia (55), where nurses most often resorted to avoidant coping strategies.

However, it should be noted that direct comparison of our results with findings made by other researchers is limited because coping strategies are not systemized worldwide. Most studies conducted in populations of nurses addressed strategies oriented towards active coping with stress. These strategies aimed at the identification of stressors, seeking information, multidisciplinary solutions, analysis of gains and losses, actions aimed at modification of the existing situation, or learning new skills (56, 57). Among avoidant strategies, other researchers reported reluctance to go to work, venting anger at the co-workers or family, excessive consumption of coffee, tea, alcohol, or using the sedatives, seeking the company of other people, denying the existing facts to distort reality, and also distancing, avoidance, selective attention, blaming, seeking social support, physical exercise or meditation (58). Support-seeking and emotion-oriented strategies include: distancing from problems, avoiding stressful events, and emotional and behavioural self-control.

Our results prove that nurses in stressful situations are willing to look for rational and positive solutions. It is most desirable to follow strategies of active coping with stress since they help to maintain good mental and physical health. Emotion-oriented and avoidant strategies have a strong negative impact on mental health and well-being. Data supporting this claim have been provided from studies carried out in the USA (40), Australia and New Zealand (50) as well as Brazil (59).

The majority of surveyed nurses have sleep problems. About a one third of them suffered from excessive daytime sleepiness and a similar number of insomnia. Prevalence of sleep problems among nurses was also reported from many studies conducted worldwide, including Norway (52), China (60) and Brazil (61). People may have sleep-related problems if they work in a very stressful environment. Many studies (62) have confirmed the negative impact of stress on the quality of sleep.

The conducted analysis revealed several significant correlations between coping strategies and sleep problems. The ESS measure was positively significant correlated with all strategies except for *active coping*. This shows that the greater the tendency to excessive sleepiness, the greater the activity in the implementation of a given coping strategy, especially in the case of avoidant and emotion-oriented strategies which were the strongest correlated. Relations between excessive sleepiness and coping strategies, in contrast to insomnia, has not been the subject of much research. Previously conducted studies reported positive correlation between excessive sleepiness and emotion-oriented stress coping strategies (63) but in contrary to our result inversed correlation with avoidant stress coping strategies (31).

Insomnia symptoms measured using the AIS questionnaire were not associated with all active strategies. The AIS measures were positively significant correlated with all avoidant strategies (except for *acceptance*) and with *venting* and *self-blame* representing emotion-oriented strategies. This shows that the greater the tendency to insomnia, the greater the activity in the implementation of all avoidant, *venting* and *self-blame* strategies. Positive correlation between insomnia and avoidant stress coping strategies was reported by other researchers (64, 65). There are also studies reporting negative correlation between insomnia and active stress coping strategies but our result have not confirmed such relation (65).

Nurses experience excessive sleepiness most often use avoidant stress coping strategies. Analysis conducted using regression models revealed correlation between ESS on all coping strategies measures but satisfactory determination coefficient *R*^2^ was only in case of avoidant strategies: (*humour, denial, substance use, behavioural disengagement*) and *self-blame* belonging to support-seeking and emotion-oriented group. Previously conducted studies support our result (13, 36) except *self-blame*.

Measures of insomnia were correlated only with seven coping strategies and satisfactory determination coefficient *R*^2^ was only in cases of *self-blame, substance use* and *behavioural disengagement*. Researchers in previous studies reported positive correlation between insomnia and support-seeking and emotion oriented strategies (18, 37) what is consistent with our the results only in case of *self-blame*.

Nurses graduated from universities were using to a significantly lower extent the support-seeking and emotion-oriented and avoidant strategies and to a significantly higher extent the active strategy compared to nurses with inferior academic status. The type of hospital unit had an impact on how nurses cope with stress. The only regularity with this factor is usually the difference between intensive care unit nurses and other nurses. Head nurses chose significantly more often the active coping strategies than nurses themselves (66, 67). In our research, we obtained statistically significant correlations only in the case of education and only with two strategies from the avoidant group: *denial* and *behavioral disengagement* and *use of instrumental support* from support-seeking and emotion-oriented group. The correlations were statistically very weak, and in the case of *use of instrumental support* with a very low coefficient of determination.

More interesting and much better statistically parameterized results were obtained for occupational factors in interaction with ESS and AIS measures of sleep problems. Nurses with tertiary education experiencing excessive sleepiness less frequently used the strategy of *humour, behavioural disengagement* and *religion* than persons without tertiary education. Similarly those experiencing insomnia but in case of *humour, substance use* and *religion*. Having a tertiary education discourages nurses from escaping into *religion* coping strategy regardless of the type of sleep problem.

Nurses working in interventional wards experiencing excessive sleepiness used the strategy of *humour, religion* and *positive reframing* less often than those working in conservative wards but at the same time those suffering from insomnia used the strategy of *humour* more often than those working in conservative wards. The reason of such relationship could be that nurses working in interventional wards are exposed to greater stress and excessive sleepiness is associated with adopting avoidant strategies like *humour* and *religion* (13, 36). To resolve this issue, it would be necessary to extend the research with additional psychometric measures. The most interesting result seems to be the influence of the type of hospital ward on undertaking *humour* strategy (one of the avoidant strategies), which is completely different depending on the suffering of insomnia or excessive sleepiness.

Age and frequency of taking sick leaves (except very weak influence on *active coping*) independently and in interaction with sleep problems measures were not statistically significant correlated with any stress coping strategies. Lack of statistically significant correlations between age and coping with stress also occurred in other studies (68).

The survey for this study was completed 2 months before the first Sars-Cov-2 infection in Poland was identified. The Covid-19 pandemic has changed the way the entire social and professional community of nurses functions in Poland and around the world. The rules of social distancing, restrictions on movement, periods of isolation, and suspended activity of various sectors of the economy have introduced the fear of loss of income and deterioration of the quality of life. These factors directly or indirectly influenced the mental and physical well-being of nurses in the workplace and their ability to cope with stress. Nurses are most prone to mental and physical stress among medical staff, including exhaustion due to extended working hours, frustration, discrimination, negative patient behaviour and the lack of contact with their own families. During the pandemic, there is also a much greater load of quantitative and qualitative work, as well as extended working time caused by the sudden and rapid growth of patients requiring hospitalization due to symptoms suggesting the infection (69).

In the current situation of the health service, it is difficult to imagine a reduction in the intensity of stress in the workplace of nurses. Therefore, at the present time, programs to support active coping with stress and effective rest, and above all sleep, are becoming extremely important. It would be useful to study how nurses cope with stress during a pandemic despite difficulties in carrying them out.

## Conclusions

The implementation of avoidant and support-seeking and emotion-oriented stress coping strategies by nurses were associated with the sleep problems.

Tertiary education discourages nurses with sleep problems from using avoidant coping strategies (humour, substance use, behavioural disengagement) and devoting themselves to religion.

Nurses with sleep problems choose different strategies for coping with stress depending on the hospital ward they work in, but the results obtained are not sufficient to define more general patterns.

## Methodological Limitations

The sample used, study design (cross-sectional study precludes identification of causal relationships between the studied variables), self-reported style questionnaires are significant limitations of the study. The research was conducted only in a single region of Poland. The reasons for not taking part in the study by 25% of invited persons are unknown due to the manner the study was conducted.

## Data Availability Statement

The raw data supporting the conclusions of this article will be made available by the authors, without undue reservation.

## Ethics Statement

The studies involving human participants were reviewed and approved by Bioethical Commission of the Medical University of Bialystok. Written informed consent for participation was not required for this study in accordance with the national legislation and the institutional requirements.

## Author Contributions

KK: concept of the research, design of article structure, conducting of the research, review of the literature, results analysis, and writing the article. EK-K: review of the literature and review of article drafts. MS: statistical analysis. All authors contributed to the article and approved the submitted version.

## Conflict of Interest

The authors declare that the research was conducted in the absence of any commercial or financial relationships that could be construed as a potential conflict of interest.
